# T helper 2 cells in asthma

**DOI:** 10.1084/jem.20221094

**Published:** 2023-05-10

**Authors:** James A. Harker, Clare M. Lloyd

**Affiliations:** 1 National Heart and Lung Institute, Imperial College London, London, UK

## Abstract

Allergic asthma is among the most common immune-mediated diseases across the world, and type 2 immune responses are thought to be central to pathogenesis. The importance of T helper 2 (Th2) cells as central regulators of type 2 responses in asthma has, however, become less clear with the discovery of other potent innate sources of type 2 cytokines and innate mediators of inflammation such as the alarmins. This review provides an update of our current understanding of Th2 cells in human asthma, highlighting their many guises and functions in asthma, both pathogenic and regulatory, and how these are influenced by the tissue location and disease stage and severity. It also explores how biologics targeting type 2 immune pathways are impacting asthma, and how these have the potential to reveal hitherto underappreciated roles for Th2 cell in lung inflammation.

## Teaser

Harker and Lloyd review the multifaceted roles of Th2 cells in human asthma, highlighting their influence of tissue location and disease severity. They also explore how Th2 cells and their cytokines have been targeted for biologic therapies for asthma patients.

## Introduction

Asthma is a common chronic airway disease affecting over 200 million individuals globally. The hallmarks of asthma include narrowing of the airways, chronic airway and tissue inflammation, hyperplasia and hyperresponsiveness of the airway smooth muscle, and airway remodeling. These changes in the respiratory tract result in a range of symptoms, the most common of which are intermittent shortness of breath, wheeze, and cough, which are exacerbated by a range of environmental triggers, including respiratory viral infections, pollution, and inhaled allergens.

**Figure 1.**
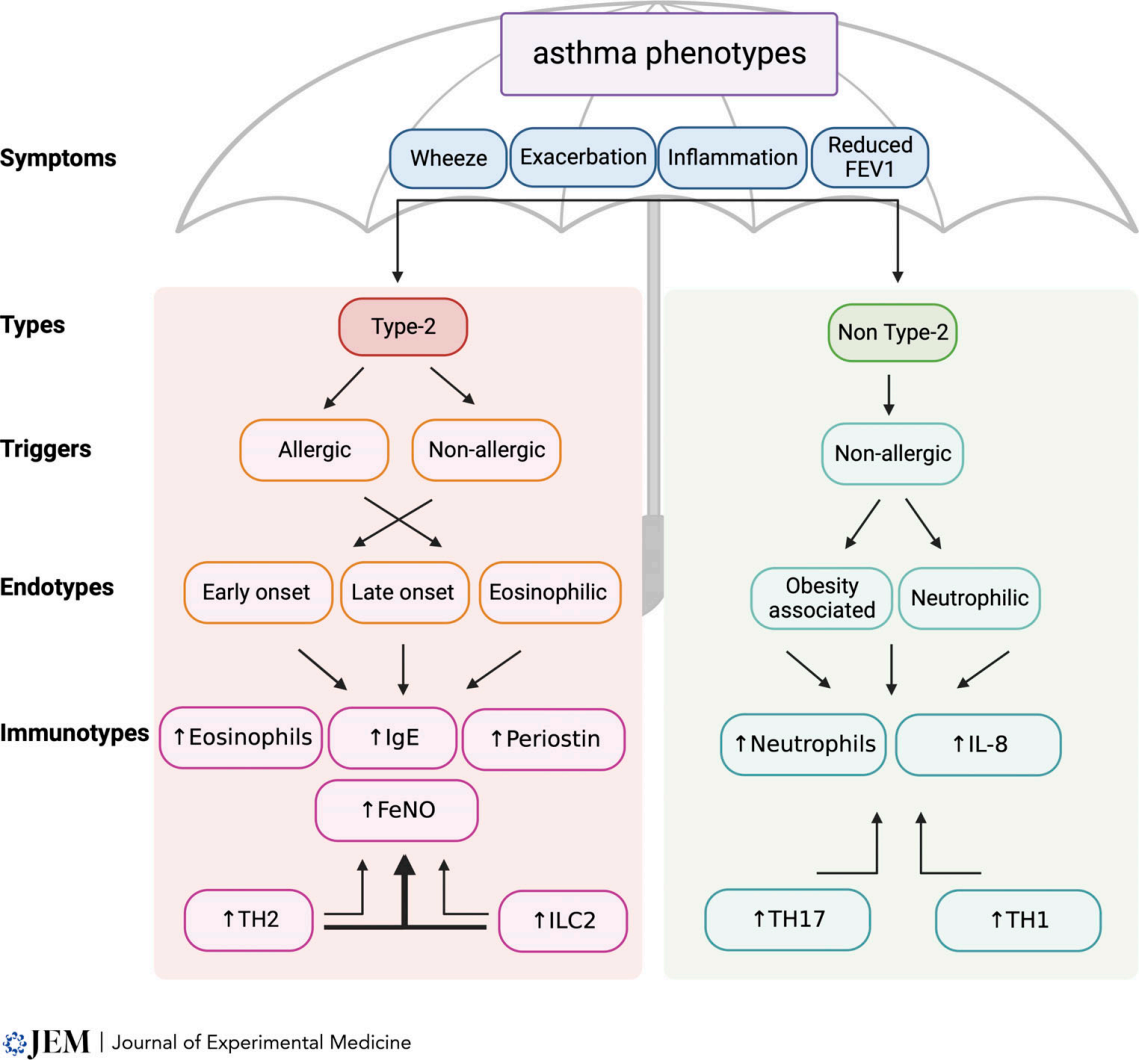
**Asthma endotypes and phenotypes. **The schematic shows the broad relationship between type 2 immune responses and asthma. Clinically diagnosed asthma is associated with a number of common “symptoms.” these can be associated with either type 2 (the majority of individuals) or non–type 2 profiles. In type 2, the most common “triggers” are allergic in origin, although other non-allergic triggers (e.g., infections, pollutants) are known. Non–type 2 triggers are predominantly non-allergic in origin. In turn, a number of “endotypes” have been described, which have one or more shared “immunotypes.” In preclinical models of type 2 asthma both ILC2s and Th2s have been shown to be capable of promoting these immunotypes, but there is limited data from humans, and it is likely these cells play somewhat overlapping, synergistic roles. An individual’s profile with regards to these features can show overlap between different aspects and fluctuate throughout life depending on age, environment, and previous or current treatments.

For many years asthma was considered a canonical type 2 disease, with atopy, eosinophilia, and elevated allergen-specific IgE being frequently observed. T helper 2 cells (Th2), as a primary source of the type 2 cytokines IL-4, IL-5, and IL-13, capable of driving all these immunological and physiological features, have therefore long been considered central to asthma pathogenesis. It is now clear however that asthma is a heterogenous disease made up of many endotypes and phenotypes including non–type 2 neutrophilic endotypes associated with obesity, smoking, and paucigranulocytic disease linked to smooth muscle dysfunction (reviewed in Wenzel, 2012 and summarized in Fig. 1). This has increased attention on non–type 2 cell types and signaling molecules and their role in asthma pathogenesis, including the contribution of Th1, Th9, and Th17 cell populations to non–type 2 asthma (reviewed in Lloyd and Hessel, 2010). However, analysis of different endotypes generally focuses on clinical symptoms associated with type 2 immunity, such as eosinophilia, serum IgE, and exhaled nitric oxide, or the general levels of type 2 cytokines, but analyses rarely reach the level of granularity such that type 2 cell populations are identified (Fig. 1). In addition, it is now accepted that the phenotype of immune cells, including T cells, within tissues may be flexible according to the surrounding cytokine milieu, local tissue pathology, and disease variations, so Th2 varieties may differ in the circulation versus the lung even within the same patient.

**Figure 2.**
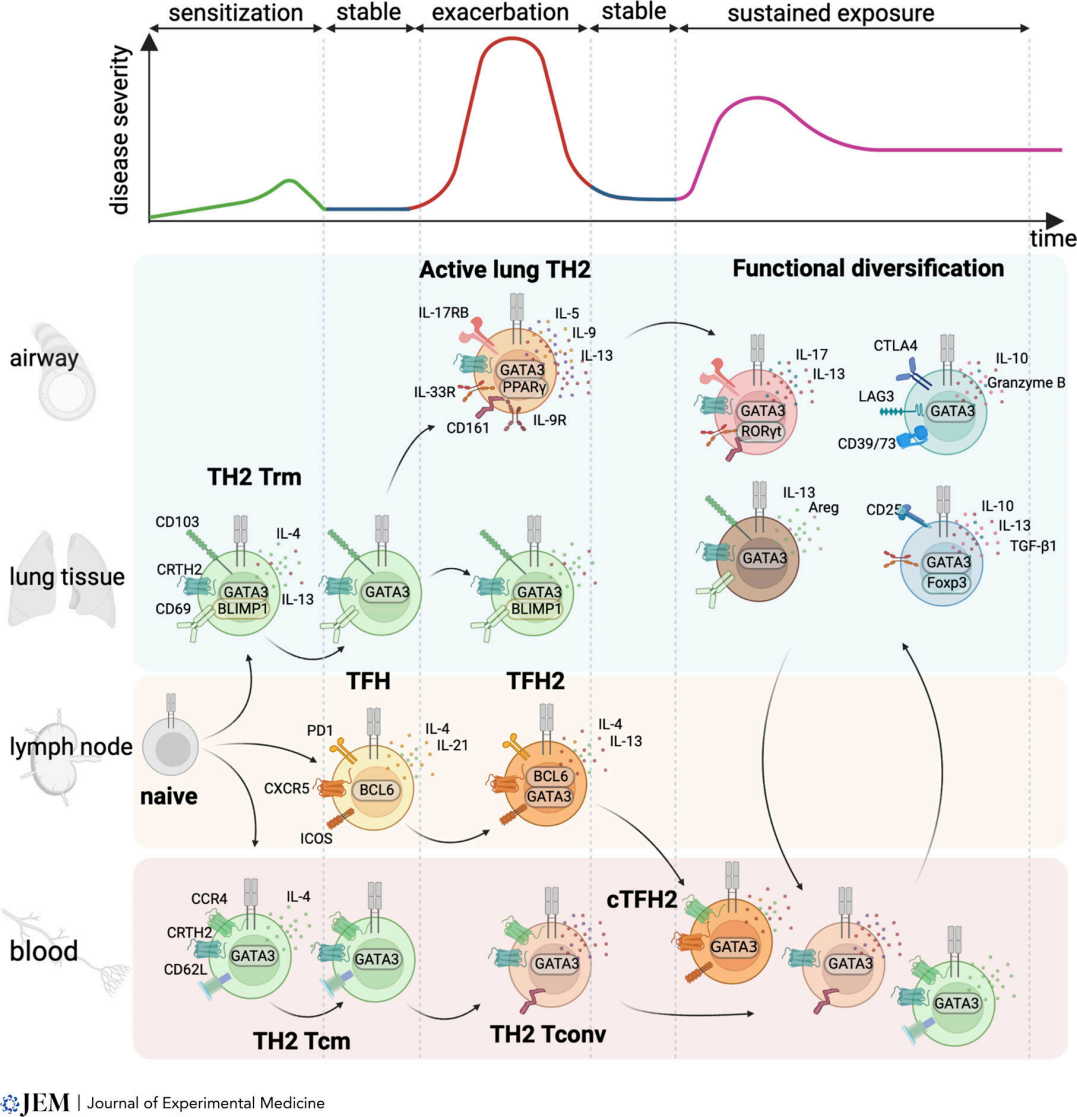
**Th2 cell diversity and dynamics during asthma inception and exacerbation.** Top: Allergic asthma displays distinct phases including (1) allergen sensitization, (2) periods of stable disease, (3) acute exacerbations, and (4) sustained enhanced symptoms. These are often linked to exposure to environmental substances (allergens, pathogens, pollutants) that drive disease. Bottom: the Th2 response to allergic asthma is heterogenous, with different mediators, surface receptors, and transcription factors expressed depending on the anatomical site and stage of disease being examined. Areg, amphiregulin.

Type 2 allergic asthma still remains the most prevalent endotype however, and is characterized by different phases of disease including (1) allergen sensitization, which primarily occurs in early life, (2) periods of stable respiratory homeostasis, (3) acute exacerbations in response to acute inflammatory stimuli, and (4) periods of chronic inflammation due to sustained exposure to environmental stimuli (See Fig. 2, top, and reviewed in Fahy, 2015). The periods of chronic inflammation, frequency of exacerbations, and therapy responsiveness differ greatly between individuals, and in search of a deeper understanding of pathophysiology, greater emphasis has been placed on non-Th2 type 2–associated cell types. The discovery of populations of innate lymphoid cells (ILCs) capable of producing type 2 cytokines (ILC2s) in an antigen-independent fashion is perhaps the most notable discovery (reviewed in Rodriguez-Rodriguez et al., 2021). ILC2s are a major source of these cytokines in murine models of allergic airways disease (AAD) and can promote many of the hallmarks of asthma in the absence of T cells (Bartemes et al., 2012; Halim et al., 2012; Klein Wolterink et al., 2012). Indeed, murine studies using protease-associated aeroallergens such as papain have highlighted that ILC2s may also be critical in the development of Th2 cells during allergen sensitization (Gold et al., 2014; Halim et al., 2014). Concurrently, the importance of epithelial-derived alarmin signaling in asthma, in particular thymic stromal lymphopoietin (TSLP), IL-25, and IL-33, has been identified (reviewed extensively including Lambrecht et al., 2019; Lloyd and Snelgrove, 2018). Polymorphisms in genes encoding these alarmins or their receptors have some of the strongest associations with an increased risk of developing asthma, and therapeutic targeting of alarmins is showing promise clinically in the treatment of allergic asthma. Indeed, damage-associated molecular patterns, TSLP, IL-25, and IL-33 are critical in initial response of the respiratory epithelium to inhaled allergens, and any subsequent inflammatory encounters within the respiratory tract with expression of the alarmin receptors can be prevalent on key immune cells including ILC2s, dendritic cells (DCs), and CD4 T cells.

The innate immune response is unequivocally pivotal in allergen sensitization of AADs. The incredible longevity of memory T cells’ functional and proliferative capacity, which can be sustained through many rounds of antigenic exposure (Soerens et al., 2023), means that once established, allergen-specific Th2 cells are a core, prominent, and potent mediator of asthma pathogenesis. Placing how Th2 cells sit within the complex immunological landscape in the human asthmatic lung has, however inevitably, become more challenging. Recent technological advances in the study of immune cell heterogeneity at the single-cell level have refocused attention on the Th2 cell, revealing hitherto underappreciated complexity and diversity of their roles in asthma. This review will highlight these new roles and reposition Th2 cells as central nexus in asthma.

## Section 1: Defining the Th2 cell in asthma

### Th2 cells of human asthma

Since the identification of GATA3 as a master transcriptional regulator that was necessary and sufficient for type 2 cytokine production by CD4 T cells (Zheng and Flavell, 1997), the Th2 cell has predominantly been described as a homogenous population of GATA3^+^, IL-4, IL-5, and IL-13 secreting CD4 T cells. The study of human Th2 cells, however, reveals the difficulty of directly translating these findings from murine models to the human disease. For instance, circulating allergen-specific Th2 cells, while more abundant in asthmatics, are routinely found in healthy individuals, thus the mere presence of Th2 cells does not, therefore, implicate them in the pathogenesis of asthma. Indeed, early sequencing analysis of circulating memory Th2 cells from asthmatics and non-asthmatics revealed that while both shared a core transcriptional and epigenetic profile, Th2 cells from asthmatics display a unique enhancer pattern and potentially pathogenic gene expression signature (Seumois et al., 2014; Seumois et al., 2016); this includes upregulated transcripts encoding proteins likely to promote survival such as *IL17RB*, which encodes the IL-25 receptor, and metabolic and apoptotic regulators such as *CPT1A* and *CASP2*, and concomitant decreases in genes such as *DUSP10*, *ZBTB10*, and *GABARAPL1* that negatively regulate T cell activation and survival via inhibition of the JNK signaling pathway, limiting IL-2 receptor signaling and promoting autophagy (Seumois et al., 2016). High-resolution single-cell RNA sequencing (scRNA-seq) of house dust mite (HDM)–specific CD4 T cells from human peripheral blood, assessed after restimulation with HDM ex vivo, confirms the enhanced expression of survival and functional factors in Th2 cells from allergic asthmatics compared not only with healthy controls but also to HDM-allergic individuals without asthma (Seumois et al., 2020). Clustering T cells identified by expression of Th2 transcriptional signature (e.g., *GATA3*, *IL4*, *IL5*, *IL13*) highlights at least two distinct clusters of Th2 cells. One of these clusters is significantly increased in HDM-sensitive asthmatics compared with HDM-sensitive non-asthmatics, and these “asthma-associated” Th2 cells are highly enriched for expression of *IL5*, *IL9*, *PPARG* (a transcription factor linked to IL-9 production), *GZMB* (encoding for the cytotoxic molecule granzyme B), *IL17RB*, and *IL1RL1* (the gene encoding IL-33R/ST2). HDM-specific Th2 cells in non-asthmatics, meanwhile, have enriched expression for *IL4*, *IL21*, and *ICOS*.

Transcriptional analysis, even at a single-cell resolution, does however have its limitations, including being limited to those genes regulated at a transcriptional level and expressed sufficiently to be identified. Use of these data in concert with other approaches is therefore key in dissecting what is taking place within a patient. Indeed, individuals with allergic disease have frequently been observed to possess different subsets of Th2 cells, commonly termed “pathogenic,” “inflammatory,” or “allergic” when analyzed at a protein level (Huang et al., 2022). In a range of allergic diseases such as nasal polyposis or eosinophilic atopic dermatitis, they have been discriminated from non-pathogenic Th2 cells using molecules including hematopoietic prostaglandin D synthase (HGPDS), CRTh2, and IL-17RB (Lam et al., 2016; Mitson-Salazar et al., 2016). The usefulness of these molecules individually to identify subsets of Th2 cells is however highly dependent on the type and severity of disease, with the majority being most robustly expressed during active disease. More recently, a subset termed Th2A cells has been identified that can be separated from conventional Th2 cells in stable disease via their coexpression of CRTh2, CD161, and CD49d, and low expression of CD27 (Wambre et al., 2017). Functionally, Th2A cells have increased expression of IL-5 and IL-9 but a similar expression of IL-4 and IL-13 compared with conventional Th2 cells, aligning with the asthma-associated cluster identified transcriptionally. Further, it is Th2A cells that appear to be specifically targeted during allergen immunotherapy in a range of conditions from food antigens such as peanuts to aeroallergens involved in asthma such as HDM (Luce et al., 2021; Wambre et al., 2017).

Taken together, there is compelling evidence for at least two distinct subsets of Th2 cells within the peripheral blood of allergic asthmatics. It is in the respiratory tract itself however that our understanding of Th2 cell biology has perhaps been most transformed with increasing recognition that tissue-resident memory T cells (Trm), rather than recruited T cells, may act as the critical mediators of respiratory health and disease (Gray and Farber, 2022). A recent scRNA-seq atlas of human bronchial biopsies revealed the presence of multiple distinct lung-resident CD4 T cell populations not found within the circulating blood (Vieira Braga et al., 2019). When comparisons were made between healthy and asthmatic tissue, asthmatics were found to exhibit tissue-resident GATA3 expressing CD4 T cells in their airway wall (Vieira Braga et al., 2019). These lung-resident Th2 cells, while transcriptionally distinct from their circulating counterparts, possess elevated expression of genes including *HGPDS*, *PPARG*, *IL17RB*, and *IL9R* linked to both pathogenic Th2path and allergic Th2A cells described in the circulation. In asthmatic individuals, these tissue-resident Th2 cells become the dominant driver of cell–cell interactions within the airway wall, communicating with epithelial cells via a range of pathways including IL-33, TSLP, epidermal growth factor receptor, and IL-13, which was linked to an IL-13–driven gene signature within both goblet and muco-ciliated epithelial cells.

Th2 cells are also increased in the bronchoalveolar lavage (BAL) of asthmatics compared with healthy individuals (Brightling et al., 2002; Cho et al., 2016; Cho et al., 2005; Message et al., 2008). The frequency of antigen-specific CD4 T cells against any specific allergen in the BAL in stable disease is low, but they rapidly increase within 24 h of allergen exposure (Cho et al., 2016). Likewise, BAL Th2 cells and the cytokines they produce also rapidly expand upon viral exacerbation with virus-specific and allergen-specific CD4 T cells, appearing to contribute to this expansion (Jackson et al., 2014; Message et al., 2008; Muehling et al., 2020). Collectively, these studies support the notion of a community of Th2 cells that are lung resident rather than recruited. Of the three canonical type 2 cytokines produced by lung-resident Th2 cells, IL-13 is the most readily detectable during stable disease (Hilvering et al., 2018; Hinks et al., 2015; Singhania et al., 2018), and likely the most important in promoting airway pathology. IL-13 signaling on human epithelial cells can directly inhibit differentiation of ciliated epithelial cells, promotes mucous-secreting cells with an altered mucus secretome, and concomitantly limits anti-viral defense genes (Jackson et al., 2020; Laoukili et al., 2001). An IL-13 gene signature is also most readily detectable in epithelial cells isolated from asthmatics and correlates with mucus within the airways following allergen exposure (Cho et al., 2016; Jackson et al., 2020; Singhania et al., 2018).

Alongside the canonical cytokines IL-4, IL-5, and IL-13, it is also clear that human Th2 cells can take on characteristics normally associated with other Th subsets in asthma. As highlighted above, for instance, there is pronounced overlap between potentially pathogenic Th2A and features found in Th9 cells, including IL-9 itself. Th9 and Th2 cells share similar differentiation and transcriptional features including a requirement for IL-4 signaling and expression of STAT6, IRF4, and GATA3 transcription factors (Kaplan, 2013). The necessity for TGF-β signaling and expression of the transcription factor PU.1 meanwhile distinguish Th9 cells from Th2 cells. PU.1 is critical in delineating murine Th9 cells from Th2 cells as it can directly interfere with GATA3 and IRF4 activity, and its overexpression in Th2 cells suppresses type 2 cytokines while promoting IL-9 secretion (Ahyi et al., 2009; Chang et al., 2009; Chang et al., 2005). In human asthma-associated CD4 T cells, *SPI1* (the gene encoding PU.1) is notably not upregulated in Th2 cells, even when analysis is focused on those cells producing IL-9 (Seumois et al., 2020; Vieira Braga et al., 2019). Instead, IL-9^+^ Th2 cells coexpress IL-5, but reduced IL-4, suggesting more subtle functional regulation (Seumois et al., 2020). In accordance with this, it has recently been proposed that PPAR-γ, a transcriptional factor also upregulated in disease-associated Th2 cells, is required and can distinguish IL-9^+^ Th2 cells that maintain robust IL-5 and IL-13 secretion from IL-9^−^ Th2 cells in humans (Micossé et al., 2019). Coproduction of IL-17 alongside Th2 cytokines by CD4 T cells has also been observed in individuals with asthma, particularly those with severe, steroid-resistant asthma (Irvin et al., 2014; Wang et al., 2010). These Th2/Th17 cells express both GATA3 and the master transcriptional factor of Th17 cells, RORγt, and are correlated with more severe airway hyperreactivity (AHR) and obstruction; their precise ontological relationship to Th2 and Th17 cells is, however, challenging to dissect in humans.

As highlighted above, the Th2 cells found in asthma display diverse molecular and cellular phenotypes. Caution should, however, be taken in treating each Th2 profile identified as distinct given the capacity of immune cells to rapidly adapt to environmental cues. Far more likely is that the different identities captured above represent the capacity of Th2 cells to dynamically change their phenotype depending on disease state and tissue site (summarized in Fig. 2 B).

### Mechanistic in vivo evaluation of Th2 cells function in asthma

Despite substantial progress, highlighted above, phenotyping the heterogeneity of Th2 cells in humans and delineating their role in asthma pathogenesis is more challenging to analyze. Fortunately, murine modeling of AAD, ranging from simplistic OVA sensitizations to complex polyallergic exposures, provides a diversity of tools with which to examine individual aspects of Th2 cells and their role in asthma pathophysiology (see text box). For instance, the primary roles of IL-4 in Th2 cell differentiation, IL-13 in mucus secretion and airway smooth muscle responses, and IL-5 in eosinophil recruitment and survival are robust across a range of AAD models (Lambrecht et al., 2019; Townsend et al., 2000). In vivo tools also allow a focus on Th2 cells specifically, for instance, T cell–derived IL-4 and IL-13, but not ILC2s, are essential for the development of airway hyperresponsiveness after early life exposure to either HDM or *Alternaria* exposure (Saglani et al., 2018). Likewise, HDM challenge of mice results in development of IL-9^+^ CD4 T cells in the lungs in a TGF-β and activin A–dependent fashion, and their adoptive transfer exacerbates AAD (Jones et al., 2012). PPAR-γ^+^ Th2 cells are also observed in the lungs of HDM-challenged mice, and T cell–specific PPAR-γ–deficient mice fail to generate IL-5 and IL-13 secreting Th2 cells and have more limited disease (Chen et al., 2017). Sustained intranasal exposure to either papain or *Aspergillus* meanwhile results in the formation of IL-17^+^ Th2 cells that persist in the lungs and promote enhanced inflammation during AAD compared to either conventional (IL-17^−^) Th2 cells or Th17 cells when adoptively transferred (Wang et al., 2010).

There are numerous protocols to generate allergic airway inflammation in mice. Historically mice were systemically sensitized with the model antigen OVA emulsified with the adjuvant Alum, followed by challenge with aerosolized OVA. This protocol induces a highly polarized type 2 pathology, with increased frequencies of these relatively homogenous Th2 cells in their lungs and airways that correlate strongly with many features of type 2 asthma including eosinophilia, T2 cytokine production, airway hyperresponsiveness, and allergen-specific IgE. More recently, models involving inhaled allergens such as HDM, ragweed, or cockroach have been adopted. When delivered using a sensitization and challenge dosing regimen, these are arguably more “clinically relevant” because they use agents that asthmatic patients are allergic to, do not involve the use of a T2 polarizing adjuvant, and use the airway route for sensitization and challenge. Chronic intermittent exposure, to mimic regular low-dose allergen exposure often experienced by humans, results in a more heterogenous lung inflammatory milieu and airway remodeling. Airway exposure to fungal allergens, such as *Aspergillus* or *Alternaria*, can also result in fibrosis alongside type 2 inflammation. Exposure to a mixture of these allergens also results in asthma pathophysiology and is important since relatively few asthma patients are mono-sensitized.

Murine models also allow the functional evaluation of genes identified by genome-wide association studies or investigated for other reasons. For instance, polymorphisms in *DENNB1*, expression of which is found in DCs, natural killer cells, and activated T cells, are linked to development of asthma (Sleiman et al., 2010). Dennb1 deficiency in mice results in a hyperallergic phenotype as a consequence of altered TCR signaling that only affected Th2 cells, and led to increased IL-4, IL-5, and IL-13 production (Yang et al., 2016). Further investigations have revealed that under normal circumstances proximal TCR signaling is maintained by the E3 ubiquitin ligases Itch and WWP2, and in their absence, TCR hypo-responsiveness leads enhanced Th2 differentiation and lung inflammation (Aki et al., 2018). In a similar vein, a CRISPR-Cas9 screen of Th2 differentiation identified expression of αvβ3 integrin as essential in both priming and polarization of Th2 cells (Szeto et al., 2023). Mouse models have also enabled the role of microenvironmental cues in Th2 differentiation to be identified such as hitherto underappreciated cytokines like IL-1β or metabolic reprogramming (Caucheteux et al., 2016; Stark et al., 2019; Yang et al., 2013). Highlighting the mechanistic benefits in vivo models can provide while re-enforcing the central role of Th2 cells in asthma.

Genetic reporters meanwhile allow location-based information of the T cell response to be evaluated. One of the most striking observations, initially made in *Nippostrongylus brasiliensis*–induced lung inflammation, is the divergent production of IL-4 and IL-13. In the lungs, IL-13^+^, IL-4^+^, and IL-13^+^/IL-4^+^ CD4 T cells are all present, with IL-13^+^ CD4 T cells being dominant (Liang et al., 2011; Prout et al., 2018). Meanwhile, T follicular helper cells (TFH) rather than Th2 is the predominant IL-4 secreting cell in the LNs while IL-13 is produced at a much lower frequency, primarily by LN-localized Th2 cells (Glatman Zaretsky et al., 2009; King and Mohrs, 2009; Uwadiae et al., 2019). This heterogeneity is reinforced by scRNA-seq of BAL CD4 T helper cells after intranasal sensitization and challenge of mice with HDM, which shows at least six distinct clusters of CD4 T cells developing in response to allergen in the airways alone (Tibbitt et al., 2019). This includes a cluster with a distinct Th2 transcriptional signature which includes *Gata3*, *Il5* and *Il13*, *Pparg*, *Cd200r1* and *Il6*, and a dependence on lipid metabolism but is temporally separate from *Il4* expression, aligning with observations of the pathogenic Th2 population seen in patients.

Asthma results in both systemic and local inflammation with parenchymal, perivascular, and airway inflammation all observed in murine models of AAD (Johnson et al., 2004), allowing the importance of location in determining the function of Th2 cells to be assessed. Allergen-specific Th2 Trm cells rapidly develop and persist in the lungs of mice after HDM exposure (Hondowicz et al., 2016). These Th2 Trm are necessary and sufficient for airway hyperresponsiveness and type 2 inflammation on allergen challenge and depend on IL-2 signaling for their development and tissue retention. Once present, these Th2 Trm rapidly react to allergens and direct early inflammatory responses (Turner et al., 2018). Th2 Trm are dependent on the transcription factor Blimp-1, unlike Th2 cells generated via systemic allergen challenge, which is upregulated in an IL-10–STAT3–Blimp-1 dependent fashion to promote Gata3 expression (He et al., 2020). While Th2 Trm are therefore critical in driving airway inflammation, mucus metaplasia, and airway hyperresponsiveness, it is not, however, that circulating Th2 cells are irrelevant in AAD. Indeed, a recent study identified a non-redundant role for circulating Th2 cells in promoting parenchymal and perivascular inflammation after aeroallergen challenge (Rahimi et al., 2020).

Even once resident, mouse models have shown that Th2 cells are highly influenced by their microenvironment. The level of IL-33/ST-2 signaling appears particularly critical. IL-33 signaling on Th2 cells promotes the acquisition of a proinflammatory memory state, allowing elevated expression of IL-5 (Endo et al., 2015). Mechanistically, pathogenic tissue-resident Th2 cells increase expression of acetyl-CoA carboxylase 1, a key regulator of fatty acid biosynthesis, leading to upregulation of IL-33R, increased sensitivity to IL-33, and elevated IL-5 production (Nakajima et al., 2021). IL-33 signaling following HDM exposure can also result in production of the epidermal growth factor receptor ligand amphiregulin by Th2 cells, reprogramming eosinophils to acquire a profibrotic state in a process distinct from that of IL-5 and enhancing airways disease (Morimoto et al., 2018). In more prolonged airway inflammation, such as that established by chronic exposure of mice to *Aspergillus,* extensive lung fibrosis is observed alongside inflammation and AHR, and Trm CD4 T cells colocalize to areas of fibrosis, express IL-5 and IL-13, and exhibit a profibrotic gene signature (Ichikawa et al., 2019). During quiescent phases, Trm have been shown to occupy distinct anatomical niches to their recruited counterparts with murine models, initially of infection, highlighting their presence in the tissue surrounding the airways (Turner et al., 2014). At an even higher resolution, IL-5–producing Trm, formed after *N. brasiliensis* or papain exposure, have recently been shown to colocalize alongside ILC2s at adventitial cuffs (Dahlgren et al., 2019) and perivascular regions surrounding intermediate to larger blood vessels, which in the lungs are primarily found proximal to airways (Dahlgren and Molofsky, 2019). These areas are enriched for immune cells alongside lymphatic and vascular flow and during type 2 responses bring Th2 cells and ILC2s into close contact with both DCs and adventitial stromal cells: fibroblast-like cells enriched for production of TSLP and IL-33 (Dahlgren et al., 2019).

## Section 2: Th2 cells as central nexus for asthma immune responses

**Figure 3.**
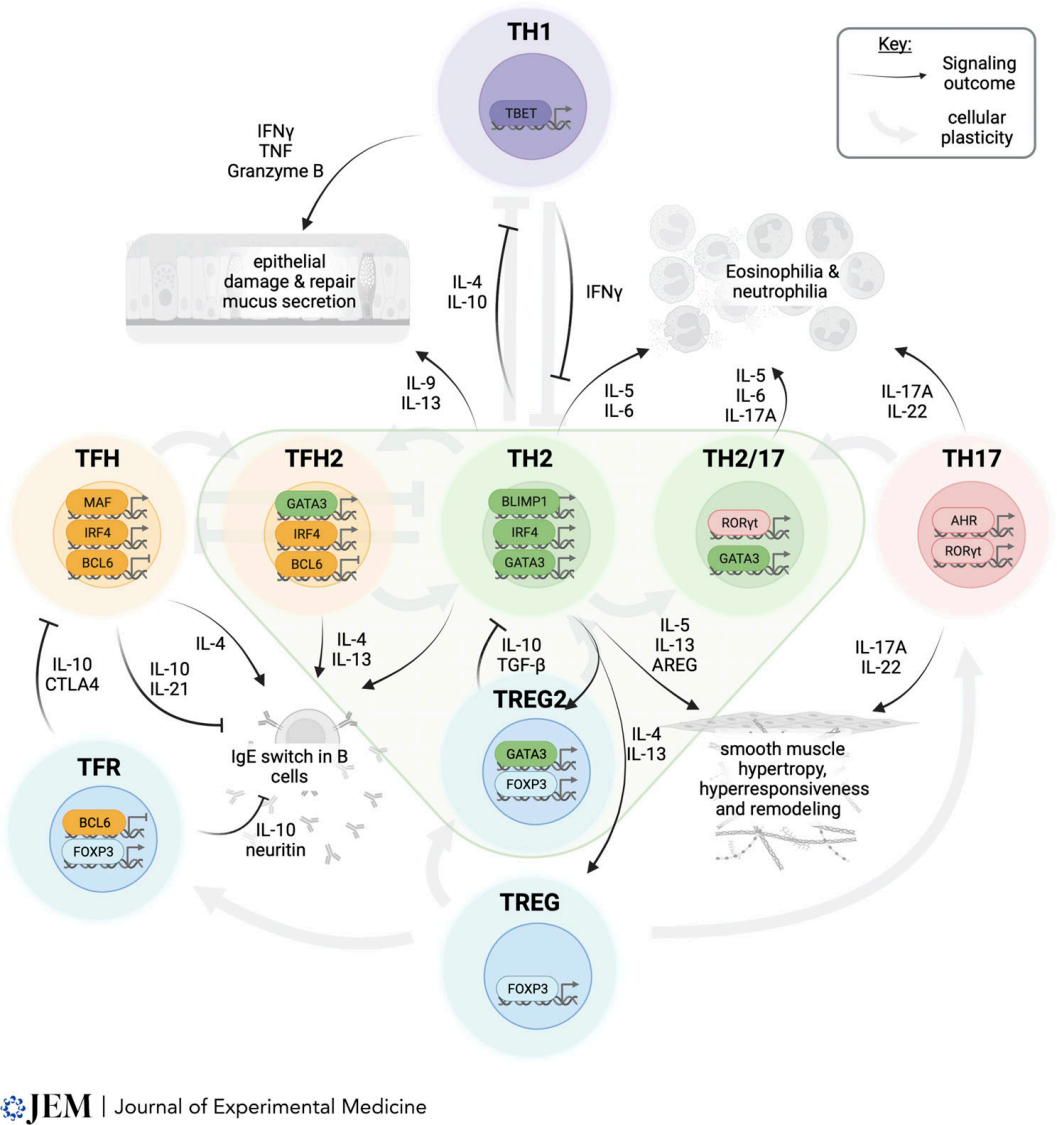
**Th2 cells as a hub for T cell responses in asthma.** Th2 cells are central contributors to asthma pathogenesis. They produce a range of soluble mediators (black arrows) that promote the pathogenic features of asthma including epithelial dysfunction, granulocyte recruitment, smooth muscle and extracellular matrix remodeling, and IgE production. They also shape the surrounding CD4 T cell response, regulating their function and identity (gray arrows). AREG, amphiregulin.

Th2 cells are the main T cells associated with pathogenesis in asthma; however, in allergic asthmatics, they remain low in number, even among allergen-specific CD4 T cells. HDM-reactive CD4 T cells isolated from peripheral blood mononuclear cells, for instance, contain CD4 T cells with Th1, Th17, and regulatory T cell (Treg)–associated transcriptional signatures, and by far the largest populations have transcriptional profiles linked to a specific activation status rather than a canonical CD4 T cell subset (Seumois et al., 2020), findings largely replicated when looking at BAL CD4 T cells found in mice after HDM challenge (Tibbitt et al., 2019). These non-Th2 T cell populations play a critical role in asthma, especially when they interact with, or functionally replace, Th2 cells (summarized in Fig. 3).

### Th1 cells

Th1 and Th2 cells are generally considered mutually antagonistic, and thus traditionally Th1 cells have been perceived as disease limiting in asthma. Fitting this during a mixed inflammatory response in the lungs, it was recently shown that IFN-γ producing Th1 topographically limits Th2 and ILC2 cells perturbing type 2 inflammation (Cautivo et al., 2022). The reality is, however, more nuanced. Th1 cells, alongside IFN-γ–secreting CD8 T cells, are readily detectable and more abundant than Th2 cells, and IFN-γ elevated in individuals with more severe asthma (Raundhal et al., 2015; Steinke et al., 2021; Wisniewski et al., 2018), where the immune profile is also more heterogenous, featuring neutrophilic alongside eosinophilic infiltration compared to those with mild allergic asthma. Both Th1 and Th2 cells also increase during viral exacerbations of asthma, with Th1 frequencies correlating with declining lung function (Muehling et al., 2020). In mouse models of mixed inflammation, such as sustained HDM exposure, inflammation is, however, usually dominated by Th2 and Th17 cells, rather than Th1 cells. If immune regulation is lost, however, for instance through deletion of IL-10 which itself is primarily produced by the Th2 and Th17 cells, Th1 cells accumulate, causing IFN-γ–dependent airway inflammation (Branchett et al., 2020). Likewise, if coupled with the bacterial product cyclic-di-GMP, HDM exposure elicits Th1 accumulation, resulting in steroid-resistant airway hyperresponsiveness (Raundhal et al., 2015). The Th1 and Th2 cell paradigm, while clearly showcasing antagonism, may highlight that in some circumstances it is the Th2 cell that is limiting a more severe Th1 cell–dependent asthma from developing. However, this requires careful analysis of lung T cells across the spectrum of human asthma phenotypes.

### Th17 cells

Aside from Th2 cells, Th17 cells and the IL-17A they produce are the most frequently observed and studied CD4 T cell populations in asthma (Newcomb and Peebles, 2013). Th17 cells and IL-17A are increased in the asthmatic airway, particularly in individuals with neutrophilic or more severe asthma (Al-Ramli et al., 2009; Irvin et al., 2014; Liu et al., 2017). IL-17A signaling on mouse or human airway smooth muscle cells enhances contractility, and while IL-13 signaling is dominant in driving airway hyperresponsive in mouse models, IL-17A signaling can operate independently of IL-13 (Kudo et al., 2012; Manni et al., 2016). There is also some evidence that Th17 cells contribute to airway remodeling in murine AAD (Zhao et al., 2013). As highlighted previously, several studies have described a population of asthmatics with a Th2/Th17 cell population expressing both RORγt and GATA3 linked to more severe disease (Irvin et al., 2014; Liu et al., 2017). Inhibition of Rorγt in a *Aspergillus oryzae*–induced mixed inflammation AAD model resulted not only in the inhibition of Th17 cells but also Th2 cells, with RORγt expression being required to suppress Bcl6, facilitating Gata3 activity, thus re-enforcing codependence between Th2 and Th17 cells in asthma (Na et al., 2018). Conversely, transcriptomics of bronchial biopsies from asthmatics indicated the presence of distinct Th2 high and Th17 high gene signatures that defined different patients suggesting reciprocal antagonism (Choy et al., 2015). A separate study found an IL-17 gene signature in the absence of an IL-13 gene signature within bronchial tissue in a subset of asthmatics with heightened neutrophilia, increased risk of exacerbations, and dysbiosis in the airways (Östling et al., 2019). A steroid-resistant Th17 endotype may develop from a steroid-sensitive Th2 endotype as a result of corticosteroid treatment. While this may be the case in some individuals, it would neither explain the observation of dual-expressing Th2/Th17 cells in some patients nor the presence of both eosinophilia and neutrophilia in many individuals.

### TFH cells

TFH are vital in the generation of T-dependent antibody responses. They are primarily found within the B cell follicles of secondary lymphoid tissue (SLO), where they interact with germinal center B cells and are critical protective antibody generation in a variety of settings, including respiratory infections (Pyle et al., 2021). Under normal conditions, TFH and Th2 cell differentiation could be seen as mutually exclusive. Bcl6, a transcriptional repressor and master regulator of TFH differentiation, and Blimp-1, highlighted above as key for Th2 differentiation, are mutually antagonistic. Bcl6 deficiency abolishes TFH differentiation while leading to accumulation of lung Th2 cells after viral or aeroallergen exposure (Hondowicz et al., 2016; Pyle et al., 2021), potentially through both cell-intrinsic and cell-extrinsic routes (Chandler et al., 2022). Likewise, IL-21, the major cytokine produced by TFH within germinal centers, suppresses IgE switch in both mouse and human B cells, even when IL-4 is also present (Ozaki et al., 2002; Suto et al., 2002; Yang et al., 2020), and TFH-derived IL-10 also suppresses IgE switch (Cañete et al., 2019). Immunodeficiencies linked to a failure to generate TFH, such as loss of function mutations in *ICOS* or *STAT3*, can also lead to heightened IgE responses despite substantially reduced concentrations of the other antibody isotypes (Bossaller et al., 2006; Grimbacher et al., 2003; Ma et al., 2008).

Despite this, in allergic diseases such as asthma, enhanced frequency of circulating TFH, surrogates of their SLO-resident counterparts, are often observed (reviewed in Varricchi et al., 2016) with allergen-specific TFH seen from an early age and enhanced frequencies associated with HDM sensitization (Foster et al., 2020). TFH, but not Th2 cells, are essential for the generation of IgE in response to aerosolized or systemic allergens, and therapeutic blockade of TFH reduces IgE and ameliorates AAD (Dolence et al., 2018; Kobayashi et al., 2017; Uwadiae et al., 2019). Indeed, TFH is the primary source of IL-4 within the SLO, a cytokine that is required for IgE switch (Meli et al., 2017; Vijayanand et al., 2012), and production of IL-4 by TFH is increased in HDM-sensitized individuals (Foster et al., 2020). TFH-producing IL-13 cells are also observed in allergen-sensitized individuals with high IgE concentrations, and IL-13 secretion by TFH is central to IgE-dependent anaphylactic responses in mice (Clement et al., 2019; Gowthaman et al., 2019). This highlights substantial functional overlap between TFH and Th2, and Gata3^+^ TFH under some conditions (Glatman Zaretsky et al., 2009), and upon adoptive transfer into HDM-sensitized mice, TFH can migrate to the lungs and differentiate into IL-4/IL-13 dual producing Th2 cells (Ballesteros-Tato et al., 2016). A population of non-Th2 IL-21–secreting CD4 T cells has also been observed in the lungs after allergen challenge, which provides essential cues to IL-21R–expressing lung-resident Th2 cells and enhances eosinophilia (Coquet et al., 2015).

In sum, TFH display substantial functional plasticity with other CD4 T cell subsets, including Th2 cells in asthma (Morita et al., 2011). Reflecting this, it has recently been proposed that TFH are grouped according to their phenotype into Group 1, 2, and 3 TFH, as is the case for ILCs (Eisenbarth et al., 2021).

### Treg cells

Tregs, particularly those expressing Foxp3, have long been known as critical suppressors of lung inflammation including allergic asthma (Lloyd and Hawrylowicz, 2009; Ray et al., 2010), and adoptive transfer of Tregs can suppress Th2-dependent AAD in mice (Kearley et al., 2005; Wilson et al., 2005). Tregs are robustly recruited in asthma though with elevated frequencies in the airways of individuals with more severe asthma (Smyth et al., 2010) and increased numbers at the same time as type 2 inflammation in response to experimental allergen challenge (Thunberg et al., 2010). In recent years, focus has shifted from analysis of Treg frequency to their phenotype and functionality during asthma. In this regard, Tregs display similar “plastic” qualities to those discussed for TFH above. Indeed, within SLOs Foxp3^+^, CD4 T cells can upregulate Bcl6 and enter the B cell follicles, where they regulate antibody responses. In murine models of allergic diseases, these T follicular regulatory cells can suppress TFH-dependent IgE through molecules like IL-10 and neuritin (Clement et al., 2019; Gonzalez-Figueroa et al., 2021; Xie et al., 2020). In barrier tissues such as the respiratory tract, meanwhile, Tregs express Gata3 and upregulate it further during inflammation (Wang et al., 2011; Wohlfert et al., 2011), where it stabilizes Foxp3 function and prevents differentiation of Tregs into effector CD4 T cells, especially Th17 cells. In homeostasis, Gata3 expression by Tregs does not result in IL-4, IL-5, and IL-13, with this being prevented by Treg expression of the E3 ubiquitin ligase Itch (Jin et al., 2013). In the lungs, however, IL-33 signaling on IL-33R (ST2) expressing Tregs results in their production of IL-13, which in this context appears to be protective against lung injury and promotes resolution of inflammation (Liu et al., 2019; Proto et al., 2018). The emergence of these Th2-like Tregs does however need to be carefully regulated to prevent their exacerbation of allergic disease, with Bcl6 and its antagonistic relationship with Blimp-1 playing a key role in balance during AAD by limiting the frequency of ST-2^+^ Tregs in the lungs (Xie et al., 2020).

### Beyond T cells

The ability of Th2 cells to influence and shape other T cell responses and the role Th2 cells and their products play in core aspects of asthma pathogenesis such as eosinophilia, airway hyperresponsiveness, goblet cell hyperplasia, and IgE-mediated inflammation is well described. They also, however, play an integral in many other processes found in asthma that have only recently come to light. For instance, their interplay and crosstalk with their innate immune partners, ILC2s, including common expression of alarmin receptors and cytokines, and DCs, with which they form close anatomical bonds, are well known (Izumi et al., 2021; Mi and Guo, 2022). They can also act as the bridge between inflammation and neuronally regulated behavioral responses, e.g., via producing CGRP to mediate nerve elongation and itching responses and causing potentiation of transient receptor potential cation channels (Meng et al., 2021; Okano et al., 2022). Th2 production of factors such as AREG, meanwhile, means they can play an intimate role in epithelial differentiation and repair upon damage (Zaiss et al., 2006). Going forward, it will be crucial to investigate these relationships in patients, translating findings from mouse models to clinical phenotypes and endotypes.

## Influence of novel biologics on Th2 immunity

The advent of biological therapies for treatment of asthma has made significant impact on the treatment choices available to patients. Given the preclinical data documenting raised type 2 immunopathology in patients and compelling data in mouse models, considerable efforts have been made to develop agents that ameliorate type 2 immune pathways. There are now multiple biological therapies available that target the type 2 mediators that Th2 and other immune cells produce, for use in patients with asthma. These in vivo human “experiments” provide an opportunity to assess the function of type 2 immune pathways in asthma.

Drugs targeting the IL-5/R axis are among the most widely used therapies for asthma treatment. Mepolizumab and reslizumab are both antibodies specific for IL-5 and are available as add-on therapies for patients with severe eosinophilic asthma. In adult patients, both have been shown to successfully reduce exacerbation rates and improve health-related quality of life, with the best results observed in patients with elevated blood eosinophil counts (Castro et al., 2015; Chupp et al., 2017; Haldar et al., 2009). Benralizumb is directed toward the IL-5R and induces antibody-dependent cell-mediated cytotoxicity whereby natural killer cells target IL-5R expressing cells such as eosinophils and basophils and elicit cytotoxic killing. It has been shown to deplete blood eosinophils and effectively reduce eosinophils in the airway lumen (sputum) and the airway mucosa (Laviolette et al., 2013). Benralizumab reduced the exacerbation rate in adult asthma patients with exacerbation-prone, severe eosinophilic asthma and improved their lung function (forced expiratory volume in 1 s [FEV1]) compared with placebo (Bleecker et al., 2016; FitzGerald et al., 2016), and has been shown to be safe and effective in long-term trials (Busse et al., 2019a; Kavanagh et al., 2021).

Dupilimab is a humanized IgG mAb designed to target the IL-4Rα chain that is common to both type 1 and type 2 IL-4R complexes. Thus, it inhibits signaling induced by both IL-4 (via IL-4Rα/γ) and IL-13 (via IL4Rα/IL13Rα). It has proved effective in downregulating type 2 immunity in a variety of disorders including atopic dermatitis and asthma. Dupilimab is licensed in a variety of countries to treat eosinophilic asthma and has been shown to reduce a number of markers of type 2 inflammation, including the chemokines eotaxin-3, TARC, and fractional exhaled nitric oxide (FeNO; Castro et al., 2018; Rabe et al., 2018; Wenzel et al., 2016; Wenzel et al., 2013). However, treatment did not reduce circulating eosinophils; in fact, some patients experienced transient hypereosinophilia, although this occurred without clinical consequences. Clinically, patients experienced a reduction in frequency of severe exacerbations, improved lung function (via an increase FEV1), and were able to lower their maintenance doses of oral glucocorticoids. The effect in clinical improvement was seen in patients who had the highest levels of circulating eosinophils going into the trials. A number of trials have tested Dupilumab in adult and pediatric asthma patients across the severity range, and outcomes were focused on clinical efficacy rather than mechanistic pathophysiological outcomes (Bacharier et al., 2021; Busse et al., 2018; Corren et al., 2020; Wechsler et al., 2022).

Preclinical studies in mouse models defined a role for IL-13 in many of the pathophysiologic features of asthma, including eosinophil recruitment, AHR, and tissue remodeling, as described by mucus hypersecretion, matrix dysregulation, and smooth muscle hyperplasia (Gour and Wills-Karp, 2015). However, the results from clinical trials in asthma patients treated with mAbs specific for IL-13 have thus far been disappointing (Nair and O’Byrne, 2019). Lebrikizumab, an IL-13 IgG4 neutralizing antibody that blocks IL-13 interactions with the IL4Rα, has so far shown only modest effectiveness. Initial trials were designed to monitor potential changes in prebronchodilator FEV1 in patients that were preselected for baseline type 2 status (according to total IgE level and blood eosinophil count) and serum periostin level (Corren et al., 2011). Periostin is an extracellular matrix protein that has been used as a surrogate biomarker for type 2 immunity, steroid responsiveness, and, perhaps, tissue remodeling (Izuhara et al., 2016). Results showed that Lebrikizumab treatment was associated with improved lung function, particularly in those that had higher levels of periostin. However, subsequent phase 3 trials failed to replicate the effect, even in patients with raised serum periostin (Hanania et al., 2016). Another IgG4 neutralizing IL-13 mAb, Tralokinumab, has also been tested in large clinical trials in severe asthma patients. An early phase 2 trial did indicate a modest improvement in FEV1 (Brightling et al., 2015). However, larger phase 2 and 3 trials failed to demonstrate consistent effects on exacerbation frequency, even when patients were preselected based on high FeNO and did not impact oral corticosteroid reduction in severe asthma patients (Busse et al., 2019b; Nair and O’Byrne, 2019; Panettieri et al., 2018). Trials of anti–IL-9 antibodies in asthma have been similarly disappointing. Although treatment was well tolerated in asthmatics across the severity spectrum (Parker et al., 2011), there was a lack of effect on either lung function, exacerbation rates, or asthma control questionnaire, even when combined with existing controller medications (Oh et al., 2013). It should be noted that these trials were conducted without patient stratification, and increasing evidence of IL-9–producing Th2 cells linked to more severe disease may warrant further investigation of anti–IL-9/IL-9R biologics.

Omalizumab is a recombinant DNA-derived mAb targeting IgE that is approved for use in children (>6 yr old) and adults with asthma. Both large-scale randomized trials and “real life” studies have shown that this treatment reduces exacerbation frequency, improves symptoms and quality of life for patients as well as facilitates reduced steroid usage (Hanania et al., 2022). Although most studies focus on the clinical efficacy, Omalizumab therapy has been shown to reduce peripheral blood eosinophilia and restore numbers of CD4^+^Foxp3^+^CD25^+^CD127^lo^ Treg, correlating with the level of asthma control (Amat et al., 2016).

Due to the association of alarmins with driving type 2 immuno-pathophysiological responses to allergens and the expression of their receptors on Th2A cells, ILC2, and other type 2 immune cells, there has been great interest in developing biologics targeting the asthma-associated alarmin triad of TSLP, IL-25, and IL-33. The hope would be to suppress both innate and adaptive pathways to provide a more comprehensive suppression of type 2 immune pathways. However, results have been disappointingly modest. Treatment of moderate to severe asthma patients with Itepekimab, a mAb targeting IL-33, improved asthma control and quality of life as well as reduced mean blood eosinophil count (Parker et al., 2011). There was no benefit to dual treatment combined with Dupilimab to block IL-33, IL-4, and IL-13. Itepekimab did reduce type 2 biomarkers such as FeNO, serum IgE, periostin, eotaxin3, and pulmonary and activation-regulated chemokine, but was less efficient than Dupilimab (Wechsler et al., 2021). Astegolimab, a selective inhibitor of the IL-33R, ST2, is safe and well tolerated and reduced annual exacerbation rate in a broad range of asthmatic patients, including those that had eosinophil low and poorly controlled severe asthma (Kelsen et al., 2021). Tezepelumab is an antibody that blocks function of TSLP, and trials have demonstrated that patients with severe uncontrolled asthma have reduced exacerbations, better asthma control, and improved health-related quality of life after receiving Tezepelumab than patients on placebo (Corren et al., 2017; Menzies-Gow et al., 2021). Interestingly, patients on Tezepelumab showed a rapid and sustained reduction in blood eosinophils, FeNO (a surrogate biomarker for inflammation), and serum IgE. A subsequent study determined that eosinophil numbers were reduced in endobronchial biopsies from patients with uncontrolled moderate-to-severe asthma following Tezepelumab, but there was no effect on other cell types examined (T cells, neutrophils, or mast cells) or on airway remodeling, as assessed by reticular basement membrane thickening and epithelial integrity (Diver et al., 2021). More recently, Tezepelumab has also been shown to reduce mucus plugging in uncontrolled moderate-to-severe asthmatic patients (Nordenmark et al., 2022).

It is clear from clinical trials of type 2 biologics that identifying the right patients contributes to the success of the treatment. The type 2 therapies seem to work most effectively in patients who are preselected based on type 2 biomarkers such as higher eosinophil counts, raised FeNO, or serum periostin. In the case of anti–IL-13 biologics, it may be necessary to use wider selection criteria—perhaps based on the most pronounced biological effects of IL-13, such as mucus production or smooth muscle cell hyperplasia (Nair and O’Byrne, 2019). Indeed mucus, specifically mucus plugging, has been proposed to be a key contributor to airflow obstruction in severe eosinophilic asthmatics (Dunican et al., 2018; Tang et al., 2022). Eosinophils are likely key in the formation of these dense mucus plugs as they are a rich source of Charcot–Leyden crystals (CLCs), formed from Galectin-10 proteins, which are readily released on activation, especially during the generation of extracellular traps (Porter et al., 2017; Ueki et al., 2018). Importantly, CLCs, and the airway damage their presence can result in, are sufficient to promote asthma-like type 2 inflammation in the lungs (Persson et al., 2019). Moreover, antibodies targeted to destabilize Galectin-10 interactions and dissolve CLCs are showing therapeutic potential in severe asthma.

An alternative strategy to neutralizing type 2 cytokines is to target the type 2 cells themselves via specific transcription factors. A randomized, double-blind, placebo-controlled, multicenter clinical trial of a novel DNA enzyme (SB010) was designed to cleave and inactivate GATA3 messenger RNA involved patients who had allergic asthma with sputum eosinophilia and who also had biphasic early and late asthmatic responses after laboratory-based allergen provocation (Krug et al., 2015). Treatment with SB010 significantly attenuated both late and early asthmatic responses after allergen provocation in patients with allergic asthma. There was also an attenuation of Th2 biomarkers such as sputum eosinophilia and tryptase (a surrogate for mast cells) and circulating IL-5 levels. However, there was no change in allergen-induced airway hyperresponsiveness and Th2 cells were not examined.

In any case, none of the agents developed so far are curative and do not replicate the efficacy of blocking type 2 pathways in animal models. This may reflect the complexity of the human disease as compared with the relative simplicity of mice, which can only mimic disease pathways rather than the complete disease. However, we lack specific knowledge of the nuances of type 2 biology in humans, particularly at the tissue level. At present, none of the human studies have examined the effect on T cell phenotypes, either in the blood or the tissue. In fact, most studies focus on effects on clinical parameters and do not include any mention of underlying mechanisms. A multiomics analysis of skin biopsies taken from atopic dermatitis patients treated with Dupilimab revealed that tissue-resident memory pathways persisted, even when clinical remission was achieved (Bangert et al., 2021). In particular, Th2A cells (CRTh2^+^CD161^+^Th cells) were found in skin tissue up to a year after clinical remission. These Th2A cells exhibited the characteristic cytokine receptor profile for the subtype, being positive for IL17RB, IL1RL1, and CRLF2, and the authors speculated that long-term maintenance of these cells within tissues would enable them to be responders to the epithelial-derived alarmins that are typical of an allergic dermatitis reaction. The persistence of these cells implies that once treatment is withdrawn, type 2 resident immune cells are ready and poised to respond to allergens and thus facilitate orchestration of allergic dermatitis pathology, leading to disease recurrence. Similar studies determining the effect of type 2 pathway biologics on tissue immune cells are urgently needed in airway inflammation.

## Conclusions

The advent of sophisticated technologies and investigative strategies to examine the human immune system has led to a greater understanding of the heterogeneous nature of immune-mediated diseases such as asthma, in particular how the Th2 cell, in its many guises, promotes and regulates various aspects of asthma pathology. Coupled with the generation of biological agents that effectively block selected cytokine/receptor pathways, the potential for enhancing our pathophysiological understanding of asthma is immense. However, clinical trials have shown that although some aspects of severe disease, most often exacerbations, are mitigated, the results have not replicated those previously observed in preclinical models. Until there is a greater emphasis on understanding how these interventions function at the tissue level in humans, the ultimate scenario of developing precision medicine strategies or furthering our understanding of type 2 immune-driven pathology at the cell and molecular level will remain out of reach.
